# Jujuboside B Inhibits Neointimal Hyperplasia and Prevents Vascular Smooth Muscle Cell Dedifferentiation, Proliferation, and Migration *via* Activation of AMPK/PPAR-γ Signaling

**DOI:** 10.3389/fphar.2021.672150

**Published:** 2021-06-24

**Authors:** Zaixiong Ji, Jiaqi Li, Jianbo Wang

**Affiliations:** Department of Interventional Radiology, Shanghai Jiao Tong University Affiliated Sixth People’s Hospital, Shanghai, China

**Keywords:** jujuboside B, neointimal hyperplasia, reactive oxygen species, proliferation, autophagy, vascular restenosis

## Abstract

The uncontrolled proliferation and migration of vascular smooth muscle cells is a critical step in the pathological process of restenosis caused by vascular intimal hyperplasia. Jujuboside B (JB) is one of the main biologically active ingredients extracted from the seeds of Zizyphus jujuba (SZJ), which has the properties of anti-platelet aggregation and reducing vascular tension. However, its effects on restenosis after vascular intervention caused by VSMCs proliferation and migration remain still unknown. Herein, we present novel data showing that JB treatment could significantly reduce the neointimal hyperplasia of balloon-damaged blood vessels in Sprague-Dawley (SD) rats. In cultured VSMCs, JB pretreatment significantly reduced cell dedifferentiation, proliferation, and migration induced by platelet-derived growth factor-BB (PDGF-BB). JB attenuated autophagy and reactive oxygen species (ROS) production stimulated by PDGF-BB. Besides, JB promoted the phosphorylation of adenosine monophosphate-activated protein kinase (AMPK) and the expression of peroxisome proliferator-activated receptor-γ (PPAR-γ). Notably, inhibition of AMPK and PPAR-γ partially reversed the ability of JB to resist the proliferation and migration of VSMCs. Taken as a whole, our findings reveal for the first time the anti-restenosis properties of JB *in vivo* and *in vitro* after the endovascular intervention. JB antagonizes PDGF-BB-induced phenotypic switch, proliferation, and migration of vascular smooth muscle cells partly through AMPK/PPAR-γ pathway. These results indicate that JB might be a promising clinical candidate drug against in-stent restenosis, which provides a reference for further research on the prevention and treatment of vascular-related diseases.

## Introduction

Vascular intervention is a very effective treatment option for the treatment of critical limb ischemia (CLI) (Baumann et al., 2014; Mohapatra et al., 2019). However, vascular restenosis caused by the destruction of the vascular barrier system has become the main disadvantage of intravascular interventional therapy for cardiovascular diseases (CVD), and has seriously affected the prognosis and quality of life of patients, which limits its long-term success (Meltzer et al., 2016). In particular, for inferior artery disease caused by diabetes, the long-term effect of the vascular intervention is even more unsatisfactory due to its small diameter and long disease duration. Although drug-eluting stents (DES) prevent restenosis, their associated delay in vascular healing and increased risk of late thrombosis make them unlikely to be a long-term solution field for restenosis (Kounis et al., 2017; Torrado et al., 2018). At present, the clinical pharmacological intervention for vascular restenosis is still unsatisfactory (Napoli et al., 2002).

Although the complex pathogenesis of intraluminal restenosis after vascular intervention has not been fully understood, it has been confirmed that excessive proliferation and migration of vascular smooth muscle cells is the leading cause of restenosis after vascular intervention (Marx et al., 2011; Carrizzo et al., 2020). Vascular intervention inevitably damages the arterial intima, and the tearing of the intima leads to collagen exposure and persistent inflammation. In the early stage, as a response to vascular injury, platelets, inflammatory cells, and vascular smooth muscle cells release growth factors such as PDGF, which can lead to the dedifferentiation of VSMCs, that is, switching from a static contraction phenotype to a synthetic phenotype (Lu et al., 2018; O’Brien et al., 1993). PDGF including PDGF-BB, PDGF-AB, PDGF-AA can bind to PDGF *α*-receptor (PDGFRα) or PDGF β-receptor (PDGFRβ) to activate PI3K/Akt, MAPK, etc., leading to the proliferation and migration of smooth muscle cells (Ma et al., 2017). Therefore, inhibiting the excessive proliferation and migration of VSMCs may help prevent neointimal hyperplasia and restenosis.

Complementary and alternative medicine (CAM), especially natural herbal products, play an important role in the treatment and management of CAD and receive more attention because of their relatively low price, fewer side effects, and multiple targets. (Rabito and Kaye, 2013; Maaliki et al., 2019; Carrizzo et al., 2020). However, it is still challenging to find more effective drugs to prevent vascular restenosis and elucidate its underlying mechanisms. SZJ is a famous traditional medicine in Asian countries, which has functions of hypnosis, calming, anti-anxiety, and anti-oxidative stress (Peng et al., 2000; Al-Reza et al., 2009; Zhang et al., 2014). JB is considered to be one of the main bioactive components in SZJ (Liu et al., 2007), and it is a natural saponin triterpenoid. Studies have shown that JB has anti-platelet aggregation effects (Seo et al., 2013), inducing tumor cell autophagy and apoptosis (Li et al., 2017; Jia et al., 2020), reducing vascular tension (Zhao et al., 2016), anti-inflammatory (Ninave and Patil, 2019). However, there are no existing studies on the effect of JB on the phenotypic transition, proliferation, and migration of vascular smooth muscle.

AMPK plays a critical regulatory role in cell energy metabolism, which is related to the increase in the AMP/ATP ratio (Lin and Hardie, 2018). The activation of AMPK can be induced by ischemia, hypoglycemia, hypoxia, etc., which may act as a sensor for cellular stress (Carling, 2017). Accumulating evidence shows that AMPK activation can inhibit the proliferation of VSMCs in damaged blood vessels and weaken the formation of neointima (Song et al., 2011; Zhang et al., 2019; Zhang et al., 2019), indicating that AMPK may be a new therapeutic target for restenosis after intervention (Xiao et al., 2018). In this study, we reported for the first time that Jujuboside B antagonizes the proliferation and migration of VSMCs induced by PDGF-BB *in vivo* and *in vitro*, mechanically, which is mediated through AMPK/PPAR-γ signaling pathway.

## Materials and Methods

### Drugs and Reagents

**FIGURE 1:**
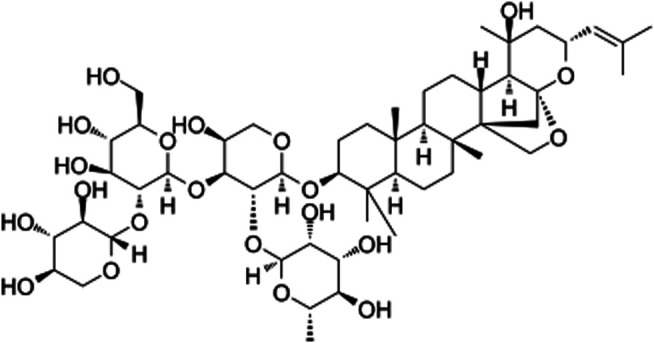
Molecular structure of Jujuboside B.

JB (chemical structure C52H84O21 shown in Figure 1, molecular weight = 1045.223, purity ≥ 98%) was brought from Shanghai Yuanye Bio-Technology Co. Ltd. (Shanghai, China). Dulbecco’s modified Eagle’s medium (DMEM), fetal bovine serum (FBS), and trypsin EDTA were purchased from Gibco BRL (Carlsbad, CA, United States ). The recombinant rat PDGF-BB was obtained from R & D Systems (Minneapolis, Minnesota, United States). Cell counting kit 8 (CCK-8) came from Beyotime Biotechnology Institute (Shanghai, China). The transwell system was from Corning Corporation (Corning, Inc., Cypress, CA). *a*-SMA, PCNA, p-AMPK, AMPK, β-tubulin antibodies were purchased from Cell Signaling Technology (Beverly, MA, United States) SM22α, OPN, PPAR-γ, LC3B, p62 antibodies were from Abcam (Cambridge, United Kingdom). GAPDH antibody and horseradish peroxidase-conjugated secondary antibodies were commercially purchased from Beyotime Biotechnology Institute (Shanghai, China). Compound C (AMPK inhibitor) and GW9662 (PPAR-γ antagonist) were purchased from MCE China (Shanghai, China).

### Ethics and Animals

The protocol of this study was approved by the Ethics Committee of the Sixth People’s Hospital affiliated with Shanghai Jiao Tong University and comply with the Guidelines for the Care and Use of Laboratory Animals published by the National Institutes of Health (NIH, 8th Edition, 2011). Male Sprague-Dawley (SD) rats (weight 200–220 g) were obtained from the Model Animal Research Center of Nanjing University (Jiangsu Province, China). In the absence of specific pathogens (SPF), all rats were housed in individually ventilated cages (three or four per cage). All rats had free access to the standard diet. All animals were housed under specific pathogen-free (SPF) circumstance which was temperature-controlled, with relative humidity (40–70%) and a 12 h/12 h light/dark cycle.

### Cell Culture

A7r5 cells (VSMCs from rat thoracic aorta) (Cell Bank of Chinese Academy of Sciences, Shanghai, China) were cultivated in DMEM, 10% FBS (Gibco, Carlsbad, United States ) in a humidified condition with 5% CO_2_ at 37°C. All cells used in the experiment were passaged 3–5 times.

### CCK-8 Assay

VSMCs were seeded in the 96-well plates at a density of 1 × 10^4^ cells per well and grown in an incubator containing 5% CO_2_ (Baumann et al., 2014) at 37°C for 24 h. When cells reached 70% confluence, the media was aspirated and cells were treated with various concentrations of JB for 24 h prior to stimulation with or without PDGF-BB (25 ng/ml). Subsequently, each well was replenished with 10 μl cell-counting kit-8 (CCK-8; Yeasen, Shanghai, China) solution and the sample was incubated at 37°C for 2 h. Finally, the cell optical density (OD) was tested at 450 nm by Molecular Devices Spectra Max i3x. Cell viability (%) was calculated based on the percentage of control. Three replicate wells were set for each group.

### Cell Counting

The VSMCs were seeded into a 6-well plate at a density of 1 × 10^5^ cells per well. After treated with PDGF-BB or JB or both, the cells were resuspended and incubated with 0.4% trypan blue dye solution. The number of cells not stained with trypan blue was measured using a Countess Automated Cell Counter (Invitrogen).

### Cell Cycle Analysis

The cell cycle was analyzed according to the protocol of the cell cycle and apoptosis analysis kit (Beyotime, Shanghai, China). VSMCs were grown to 80% confluence in a 6-well plate. After starvation for 24 h, VSMCs were pretreated by PDGF-BB or JB or both. Digested with trypsin, the cells were centrifuged (12,000 g, 5 min) and washed with 1 ml pre-cooled PBS. All samples were fixed with 75% ethanol at 4°C overnight and then washed with 1 ml of pre-cooled PBS. After cells were incubated with propidium iodide (PI) staining buffer (100 ug/mL PI and 100 ug/mL RNase A) at 37°C for 30 min, the samples were analyzed by flow cytometer (BD Biosciences, Franklin Lakes, NJ, United States).

### Annexin V and PI Double-Staining Assay

After experimental treatment, VSMCs were digested by trypsin from the six-well culture plate, collected by centrifugation, and washed with pre-cooled PBS. Annexin V and PI apoptosis kits were used for cell apoptosis analysis (BD Biosciences, United States). The cells resuspended in 100 μL 1 x binding buffer. The cells were stained with 5 ul annexin V-FITC and incubated in darkness at room temperature for 15 min. Then, the cells were stained with 5 ul PI for 5 min and resuspended in another 400 μL binding buffer. The samples were analyzed by flow cytometry (BD Biosciences, Franklin lakes, NJ, United States).

### Scratch Wound Motility Assay

Cell migration was studied using wound healing methods. VSMCs were seeded in 6-well plates (1 × 10^5^ cells per well) and grown to a concentration of 80%, then cultured in a medium containing the drug or the vehicle for 12 h. A straight scratch was made across the plate with a 200 μl pipette tip. 24 h after scratching, the wound was photographed with an inverted microscope (magnification, 100×, Leica, Germany), and the degree of cell migration was analyzed according to the percentage of recovered area with Image J software.

### Transwell Migration Assay

Transwell migration assay was used to measure the migration ability of VSMCs. Specifically, A7r5 was inoculated into the upper chamber of the transwell system (1 × 10^5^ cells per well) and treated with 100 µl of serum-free DMEM containing PDGF-BB and JB at specified concentrations. 500 µl of 10% FBS DMEM was added to the lower chamber of the 24-well transwell system. The cells were then incubated at 37°C for 24 h to migrate. After that, the non-migrating cells in the upper chamber of transwell system were removed with cotton swabs. Immediately afterwards, the transwell chamber was washed with PBS three times. The lower chamber was filled with 4% paraformaldehyde to fix the migrating smooth muscle cells. Next, the migrated cells were stained with crystal violet for 15 min. Five fields of view were randomly selected from each Transwell chamber and photographed at 100× magnification under an inverted microscope (Leica, Germany). Image J was used to perform statistical analysis on the number of migrated cells.

### ROS Measurement

The VSMCs in each group were treated accordingly and incubated with 7′-dichlorodihydro fluorescein diacetate (DCFH-DA, 10 μM) for 20 min in the dark at 37°C. The cells were washed with phosphate-buffered saline (PBS) three times, and immunofluorescence detection was performed with an inverted microscope (Leica, Germany).

### Western Blot Assay

Protein sample was harvested from cells or carotid tissues by lysis buffer. The extract was centrifuged at 4°C, 12,000 g for 20 min. Then, the supernatant was isolated and protein concentration was evaluated by a Bicinchoninic Acid (BCA) Protein Assay Kit (Beyotime Institute of Biotechnology, Shanghai, China). Equal quantities of protein were loaded and separated by sodium dodecyl sulphate-polyacrylamide gel electrophoresis (SDS-PAGE), and then transferred to polyvinylidene difluoride (PVDF) membranes (0.22, Millipore, Billerica, MA, United States). Subsequently, 5% skimmed milk in Tris-buffered saline Tween20 (TBST) was used to block non-specific binding sites and the membranes were probed with primary antibodies against PCNA, α-SMA, SM22α, OPN, AMPK, p-AMPK, PPAR-γ, LC3B, p62, GAPDH and β-tubulin (all 1: 1000 diluted) at 4°C overnight. After being thoroughly washed with TBST, the membranes were incubated with HRP-conjugated secondary antibodies (1:1000 diluted) for 1 h at room temperature. The protein bands were visualized with the enhanced chemiluminescence prime Kit (GE Healthcare, NA, United Kingdom) and photographed by a GE Amersham Imager 600 imaging system. The membranes were stripped and GAPDH or β-tubulin was used as a loading control.

### Intimal Hyperplasia Model

The rats were randomly divided into four groups: sham group, sham + JB group, injury (inj) group, injury + JB group (*n* = 6 rats/group). In the injury group and the injury + JB group, a balloon catheter was used to strip the artery to induce intimal hyperplasia of the left common carotid artery and establish a rat carotid artery injury model. Rats were anesthetized by intraperitoneal injection of 0.5% pentobarbital solution (10 ml/kg). The bifurcation of the left common carotid artery was exposed through a midline neck incision. The distal end of the external carotid artery was ligated, and the common carotid artery and internal carotid artery were clamped with hemostatic forceps. After the external carotid artery was punctured, a 1.5 F Fogarty catheter (Edwards Lifesciences, Irvine, CA, United States) was introduced into the common carotid artery (1.5 cm below the bifurcation). The balloon was inflated to 2.0 atm, and repeatedly twitched back and forth 3 times in the vessel lumen. The balloon was then deflated, the catheter was withdrawn, and the proximal part of the puncture was ligated. A palpable carotid artery pulsation was observed in all rats to ensure the restoration of blood flow. The neck incision was sutured. After balloon injury, JB (1.0 mg/kg/day) was intravenously injected for 14 days. Simultaneously, the control rats received an equal volume of phosphate buffered saline (PBS). To prepare sham-operated rats, the left common carotid artery and external carotid artery were exposed as described above, but the catheter was not inserted into the blood vessel.

### Histopathological Staining

14 days after the balloon injury, the right atrium of the rat was incised after anesthesia and the circulating blood was expelled. Then, the left ventricle was inserted with a needle and perfused *in situ* with phosphate-buffered saline (PBS) under a pressure of 90 mmHg. Paraformaldehyde (4%) in PBS was perfused *in situ* for 5 min for arterial fixation. After being incubated in 4% paraformaldehyde for 24 h, The two common carotid arteries s were embedded in paraffin. Paraffin sections (3–5 μm) were dewaxed in xylene, stained with hematoxylin and eosin (Sigma Aldrich, United States). The area of intima, media, and the ratio of intima to media (I/M) were measured with ImageJ to evaluate the neointimal hyperplasia. Four discontinuous sections from each blood vessel were measured in rats.

### Evans Blue Staining

Evans blue staining was applied to examine the reendothelialization rate of damaged blood vessels after 2 weeks. The treatment of animals in each group was as described above. Fifteen minutes before sacrifice, SD rats were injected intravenously with normal saline containing 5% Evans blue dye (Sigma-Aldrich, St. Louis, Missouri, United States). Then the rats were perfused with 4% paraformaldehyde for 10 min. After the collected left common carotid artery was longitudinally cut open, the samples were photographed with a stereomicroscope. The area stained blue is defined as the area without endothelial coverage, which is quantified by Image J software.

### Statistical Analysis

Data reproduced at least three independent experiments were expressed as mean ± SD. Statistical analysis was performed by GraphPad Prism 8 (GraphPad Software, United States). Differences among multiple groups were assessed by one-way analysis of variance (ANOVA) followed by Tukey’s test. A Student's t-test was used to compare only two groups. The significance level was accepted when *p* values < 0.05.

## Results

### JB Reduced Intimal Hyperplasia in Rats with Carotid Artery Balloon Injury

**FIGURE 2:**
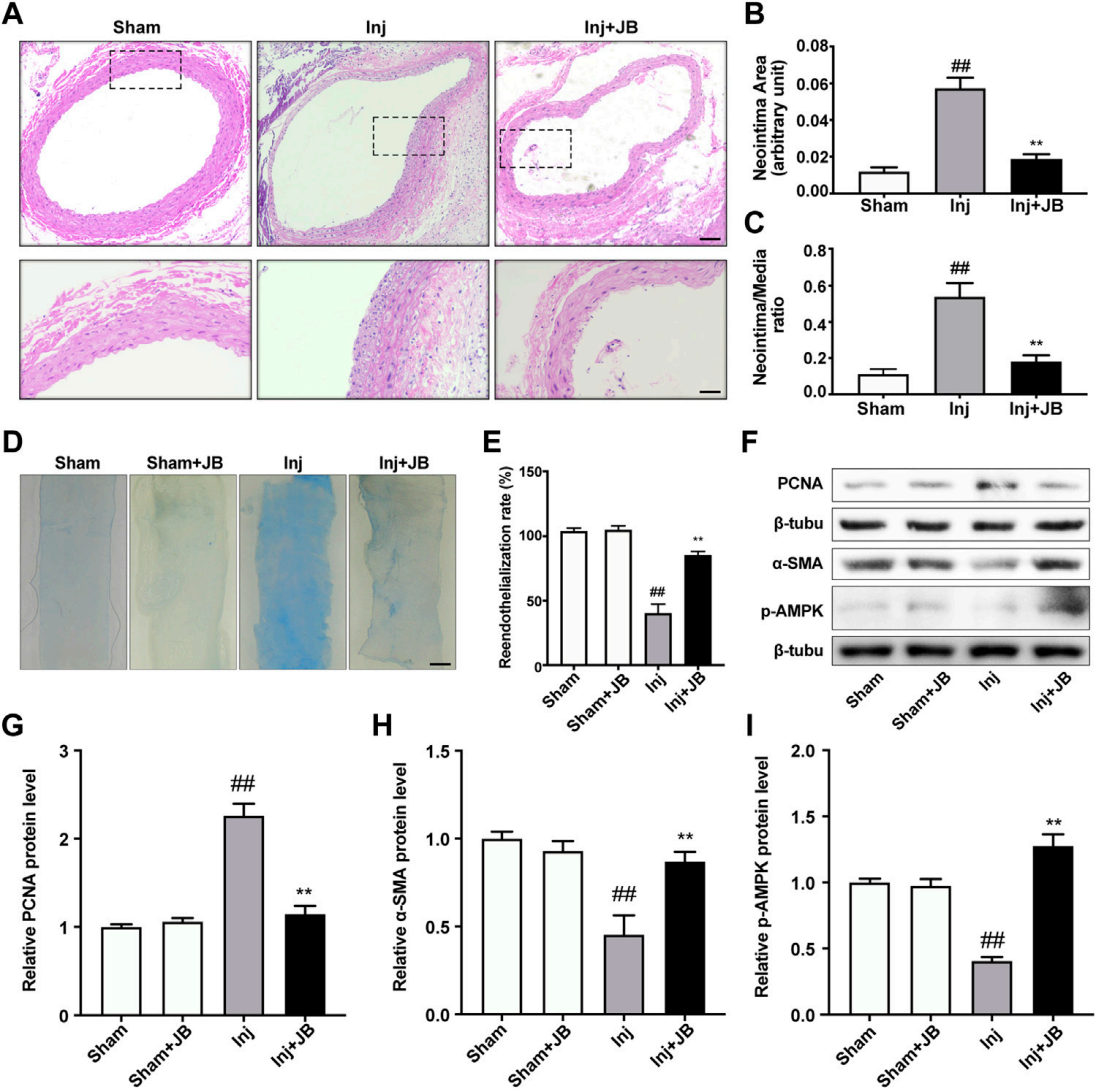
Therapeutic effect of JB on restenosis after balloon injury *in vivo*. **(A)** Representative section images of the left common carotid artery in sham-operated rats and balloon-injured rats on day 14 after operation with or without JB treatment. Scale bar: 100 μm (the upper panel), 50 μm (the lower panel). **(B,C)** Quantitative study on the area of the intima and the ratio of intima to medium (I/M) of left common carotid artery in SD rats. **(D)** Evans blue staining images of left common carotid artery in sham operated rats and balloon injured rats on the 14th day after operation. Scale bar: 1 mm. **(E)** Comparison of reendothelialization rate of left common carotid artery in each group. **(F)** Western blotting analysis of PCNA, α-SMA and *p*-AMPK in left common carotid artery of sham-operated rats and balloon-injured rats on the 14th day after operation with or without JB treatment. **(G–I)** Quantification of PCNA **(G)**, α-SMA **(H)**, p-AMPK **(I)** protein expression levels between groups. Data are expressed as mean ± S.D. ^##^ *p* < 0.01 vs. Sham group. ^**^ *p* < 0.01 vs. Inj group. *n* = 6.

To explore the effect of JB on restenosis after carotid balloon injury *in vivo*, histopathological staining was used to examine the impact of JB on neointima hyperplasia in rats with carotid balloon injury *in vivo*. After 14 days of JB treatment, we observed that balloon injury significantly increased neointimal area and intima/media (I/M) ratio, while JB treatment significantly decreased neointimal area and I/M ratio (Figures 2A–C). Also, Evans blue staining was used to detect the effect of JB on the reendothelialization of blood vessels after injury. As shown in the Figures 2D,E, JB significantly promoted the reendothelialization of blood vessels after balloon injury.

Besides, Western blotting analysis was performed in the left common carotid artery to further evaluate the effect of JB on the proliferation, migration and phenotype transformation of VSMCs *in vivo*. As shown in the Figures 2F,G, the relative protein expression level of PCNA in the balloon injured rats treated with JB was significantly lower than that in the injury group. α-SMA is a representative marker of contractile VSMCs, and its expression was relatively high in the sham group (Figure 2F). In the injury group, VSMCs were transformed into a synthetic phenotype with low expression levels of *α*-SMA (Figure 2H). Notably, the relative protein expression level of α-SMA in JB-treated balloon injured rats was significantly higher than that in the injury group. Furthermore, to explore the mechanism of JB, we investigated whether JB enhanced activation of AMPK *in vivo*. Common carotid artery ligation significantly reduced the phosphorylation level of AMPK, while the administration of JB significantly increased the phosphorylation level of AMPK (Figures 2F,I). These data suggested that JB attenuated neointimal hyperplasia by promoting reendothelialization and reducing VSMCs proliferation and migration after carotid balloon injury.

### JB Inhibited PDGF-BB-Induced Phenotype Transition of VSMC *in vitro*

**FIGURE 3:**
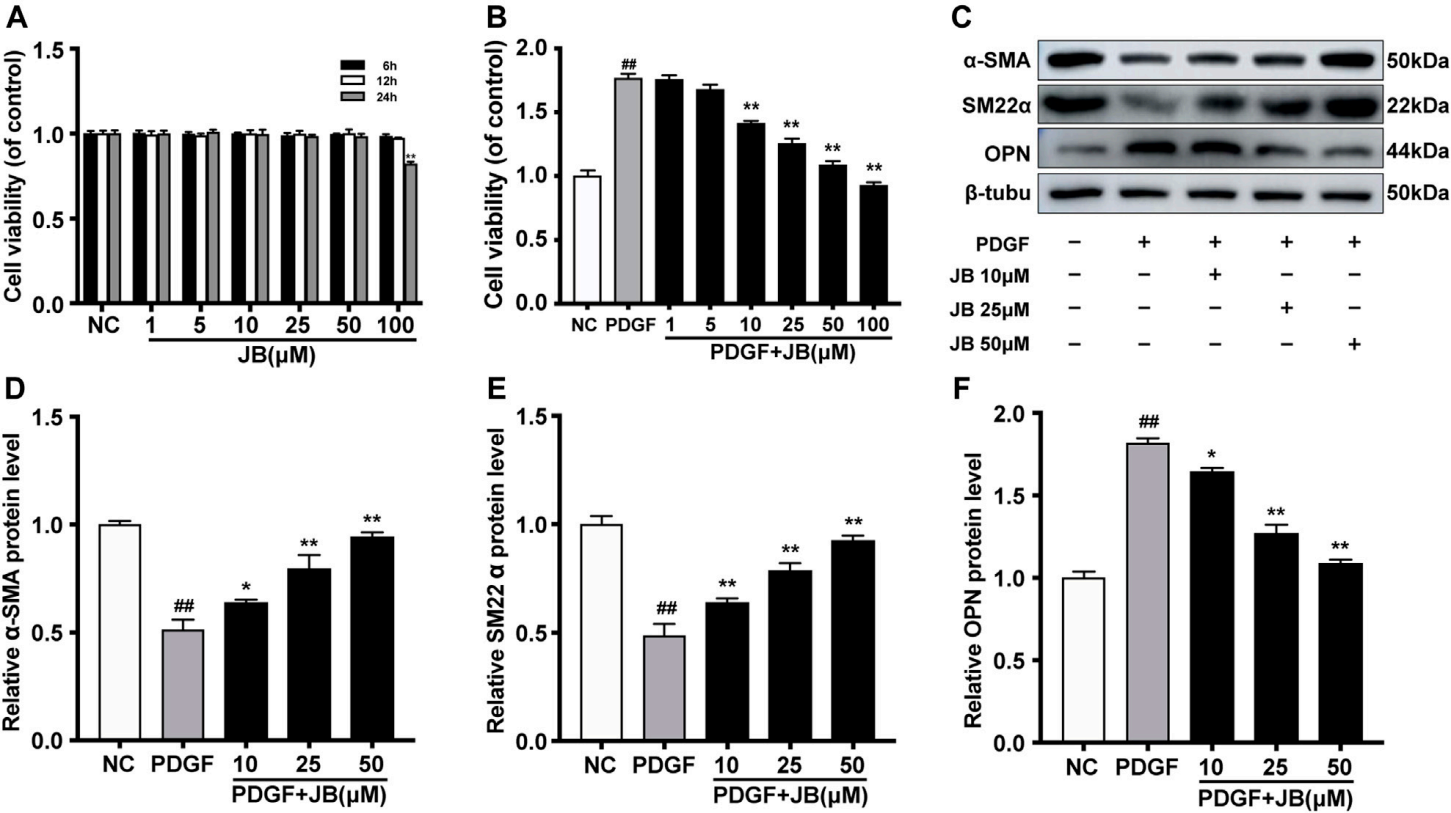
JB abolished PDGF-BB induced dedifferentiation of VSMCs. **(A)** Effects of different concentrations of JB treatment on the vitality of VSMCs according to CCK-8 assay. **(B)** Effects of JB treatment on the viability of VSMCs induced by PDGF-BB. **(C)** Western blotting analysis of α-SMA, SM22α and OPN in VSMCs stimulated by PDGF-BB and pretreated with different concentrations of JB. **(D–F)** Quantification of α-SMA **(D)**, SM22α **(E)**, OPN **(F)** protein expression levels between groups. Data are expressed as mean ± S.D. ^##^ *p* < 0.01 vs. Control. *p < 0.05 and **p < 0.01 vs. PDGF-BB group. *n* = 3.

As shown in Figure 3A, the cytotoxic effect of JB on A7r5 was studied. The survival ability of VSMCs was significantly inhibited by 100 μM JB in a time-dependent manner. Therefore, the concentration of 10, 25, and 50 μM JB was used to pretreat A7r5 for 12 h, to exclude the effect of cytotoxicity. To test the effect of JB on PDGF-BB-induced A7r5 phenotype transformation, VSMCs were pretreated and different concentrations of JB for 12 h (10, 25, and 50 μM), and then stimulated with PDGF-BB for 24 h. As shown in Figures 3C–F, PDGF-BB (25 ng/ml) promoted the transformation of the A7r5 phenotype from contractile phenotype to synthetic phenotype, which was confirmed by the increased expression of VSMCs synthetic gene OPN and the decreased expression of contractile genes *α*-SMA and SM22α (Figure 3C). JB pretreatment inhibited PDGF-BB-induced dedifferentiation of A7r5 in a dose-dependent pattern.

### JB Inhibited VSMCs Proliferation Induced by PDGF-BB

**FIGURE 4:**
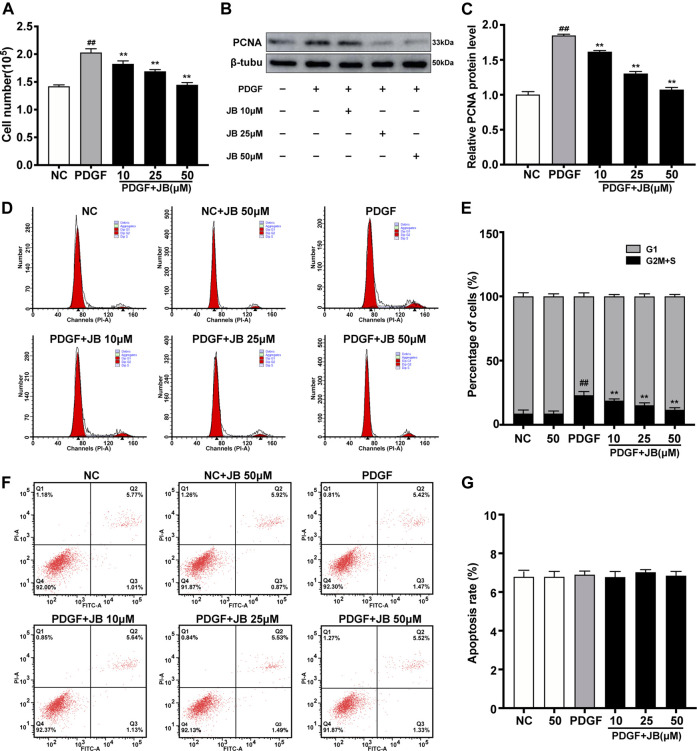
JB inhibited the proliferation of VSMCs induced by PDGF-BB. VSMCs were incubated with PDGF-BB in the presence of different concentrations of JB. **(A)** Evaluation of the proliferation of VSMCs by direct cell counting. **(B)** Western blotting analysis of PCNA protein expression. **(C)** Quantification of the relative protein level of PCNA. **(D)** Representative cell cycle images showing the cell cycle progression of VSMCs between treatment groups. **(E)** Quantitative analysis of cell cycle distribution in each group. **(F)** Determination of Annexin V-PI of VSMCs in each group. **(G)** Quantitative analysis of the percentage of apoptotic cells by flow cytometry. Data are expressed as mean ± S.D. ^##^ *p* < 0.01 vs. Control. **p < 0.01 vs. PDGF-BB group. *n* = 3.

As shown in Figure 4A, the cell counting assay was used to examine the effect of JB on PDGF-BB-induced proliferation of A7r5. Compared with the control group, PDGF-BB significantly increased the number of VSMCs. However, JB pretreatment significantly reduced the number of VSMCs in a dose-dependent manner (10, 25, and 50 μM). Next, as shown in Figure 3B, the CCK-8 experiment was used to further analyze the inhibitory effect of JB on VSMCs. Compared with the normal control (NC) group, the cell viability was significantly increased after treatment with PDGF-BB. However, JB pretreatment of 10–50 μM significantly reduced cell viability. Besides, the protein expression level of proliferation markers, proliferating cell nuclear antigen (PCNA) was detected by Western blotting analysis. As shown in Figures 4B,C, PDGF-BB significantly induced PCNA expression. However, compared with the PDGF group, JB pretreatment significantly decreased PCNA protein expression in a concentration-dependent pattern.

Cell proliferation is strictly controlled by the progression of the cell cycle. Therefore, we studied the influence of JB on the cell cycle progression by flow cytometry. As shown in Figures 4D,E, PDGF-BB significantly increased the proportion of cells in the S phase compared with the NC group. However, after PDGF-BB stimulation for 24 h with JB pretreatment for 12 h, the proportion of cells in the S phase was significantly decreased compared with the PDGF group. The decrease in cell number might be due to inhibition of cell growth or induction of apoptosis. To investigate whether JB induced PDGF-BB-stimulated A7r5 apoptosis, Annexin V and PI double-staining assay were performed. As shown in Figures 4F,G, there is no significant difference between the groups, which indicated that the inhibitory effect of JB on PDGF-BB stimulated A7r5 has nothing to do with apoptosis.

### JB Inhibited VSMCs Migration Induced by PDGF-BB

**FIGURE 5:**
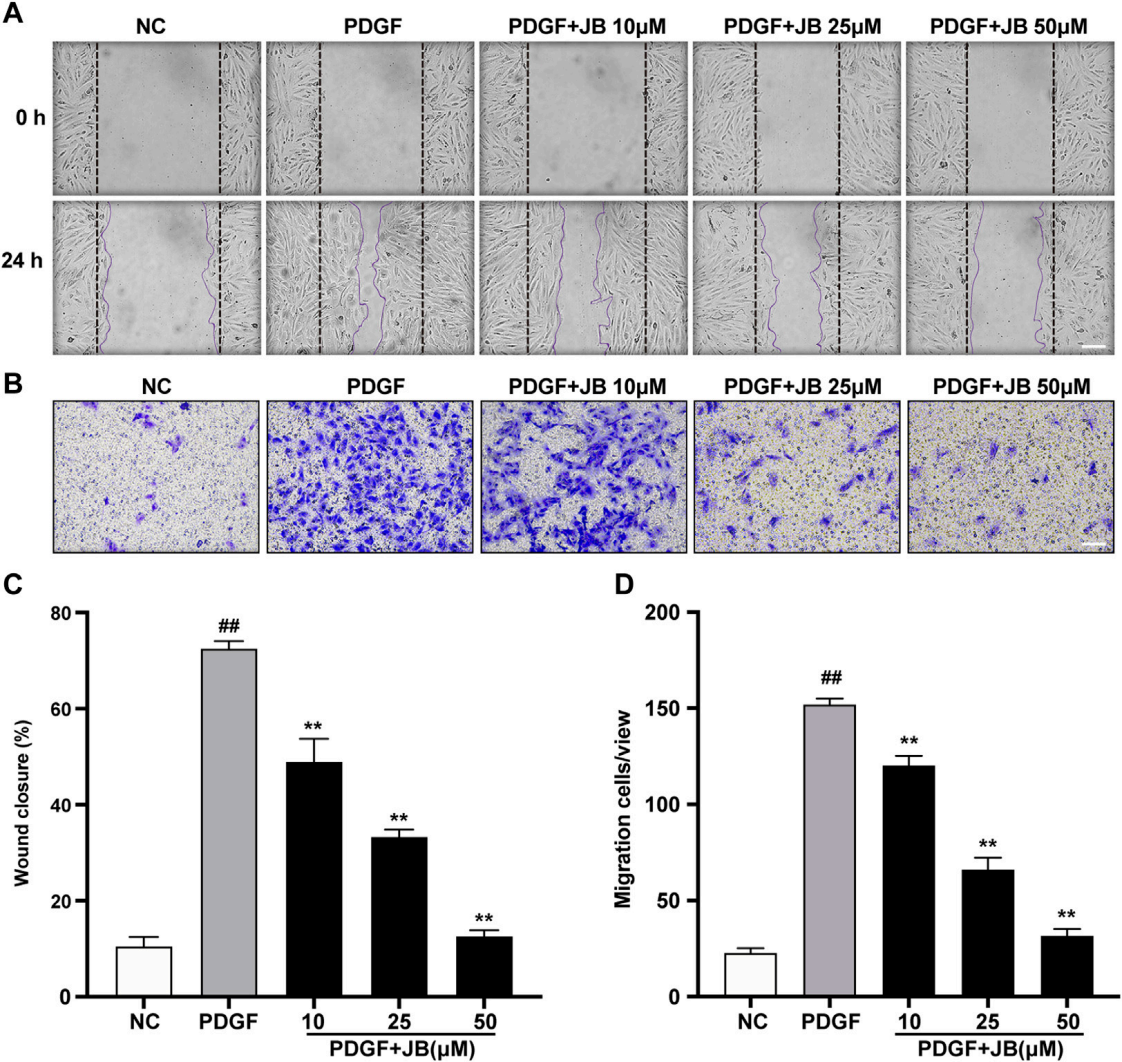
JB inhibited the migration of VSMCs induced by PDGF-BB. **(A,C)** Representative images of cell migration by wound healing test **(A)** and quantification of wound healing in each group **(C)**. Scale bar: 100 μm. **(B,D)** Representative images of cell migration by Transwell assay **(B)** and quantification of VSMCs stained with crystal violet in each treatment group **(D)**. Scale bar: 100 μm. Data are expressed as mean ± S.D. ^##^ *p* < 0.01 vs. Control. **p < 0.01 vs. PDGF-BB group. *n* = 3.

Scratch wound motility assay was used to examine the effect of JB on the migration of VSMCs *in vitro*. As shown in Figures 5A,C, 25 ng/ml PDGF significantly reduced the wound opening due to the increased migration of VSMCs. Compared with the PDGF group, pretreatment with 10, 25, and 50 μM JB significantly inhibited the migration of VSMCs induced by PDGF in a concentration-dependent manner. Next, to further study the effect of JB on VSMCs migration, a Transwell analysis was carried out. As shown in Figures 5B,D, consistently, JB significantly inhibited the migration of A7r5 cells in a dose-dependent pattern.

### JB Attenuated VSMCs Autophagy and ROS Generation Promoted by PDGF-BB

**FIGURE 6:**
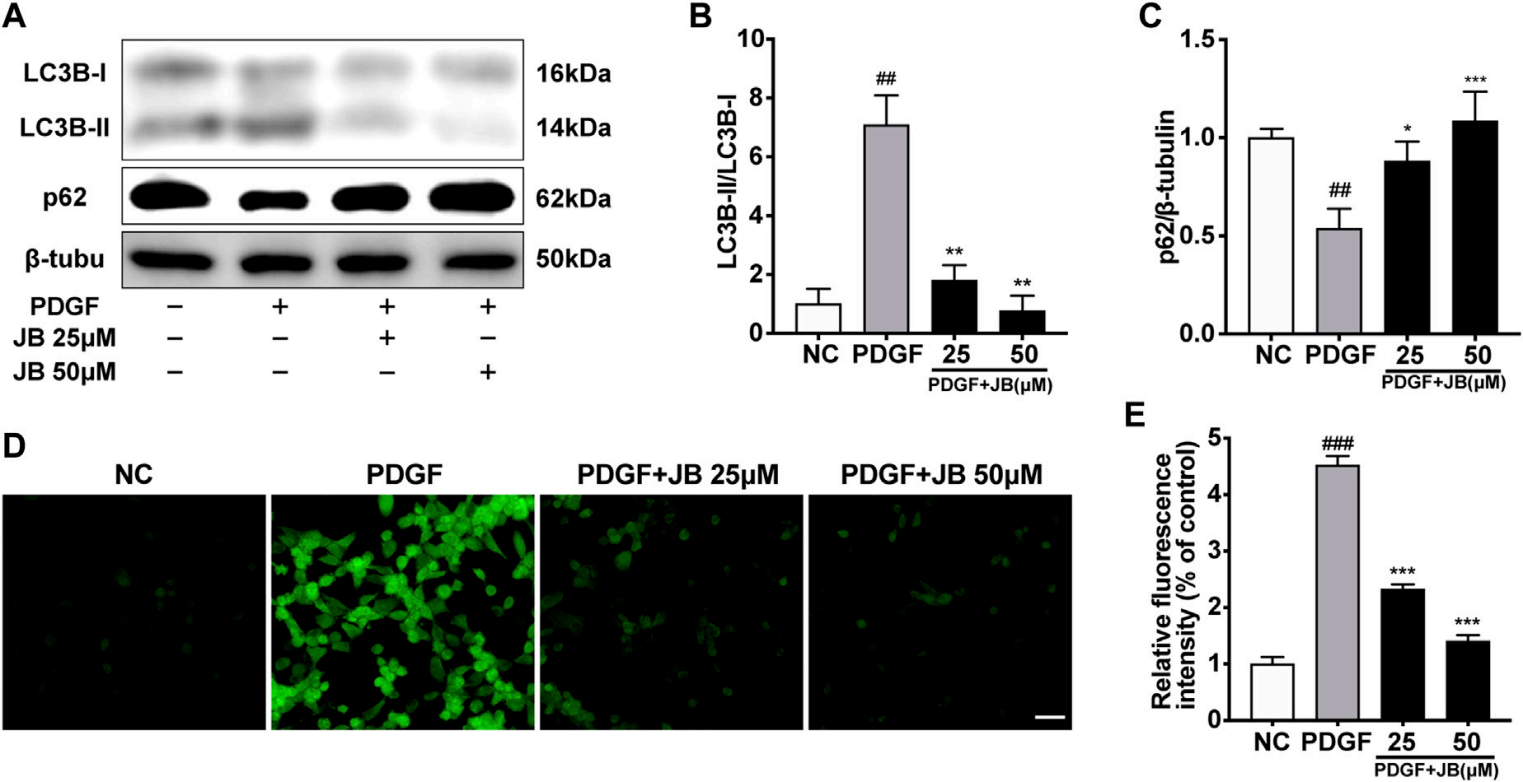
JB attenuated the autophagy and ROS generation evoked by PDGF-BB in VSMCs. **(A)** Western blotting analysis of LC3B, p62 in VSMCs. **(B,C)** Quantification of LC3B-II/I **(B)**, p62 **(C)** protein expression levels between groups. **(D,E)** ROS generation was evaluated by DCFH-DA fluorescence **(D)** and quantification of relative ROS fluorescence intensity **(E)**. Scale bar: 100 μm. Data are expressed as mean ± S.D. ^##^ *p* < 0.01 and ^###^ *p* < 0.001 vs. Control. *p < 0.05, **p < 0.01 and ***p < 0.001 vs. PDGF-BB group. *n* = 3.

To investigate whether PDGF-BB-induced cellular autophagy is alleviated by JB, we tested the expression of LC3B II/I and p62, because the up-regulation of LC3 B II/I and the degradation of p62 fully reflect the induction of autophagy (Tai et al., 2016). The results showed that *in vitro*, compared with the control group, PDGF-BB treatment increased the expression ratio of LC3 B II/I and decreased the expression level of p62 (Figures 6A–C). Besides, compared with the PDGF-BB group, JB significantly down-regulated the expression of LC3 B II/I and upregulated the expression level of p62, which indicates that JB can inhibit autophagy activated by PDGF-BB. Under certain pathological conditions, oxidative stress may be related to autophagy (Salabei et al., 2013), so we also tested the effect of JB on reactive oxygen species (ROS) generation in VSMCs. As shown in Figures 6D,E, PDGF-BB caused a significant increase in green fluorescence. It is worth noting that this effect was inhibited by JB treatment. Taken together, these results indicate that JB treatment could reduce PDGF-BB-mediated autophagy and ROS accumulation in VSMCs.

### JB Regulated AMPK/PPAR-γ Signaling Pathway

**FIGURE 7:**
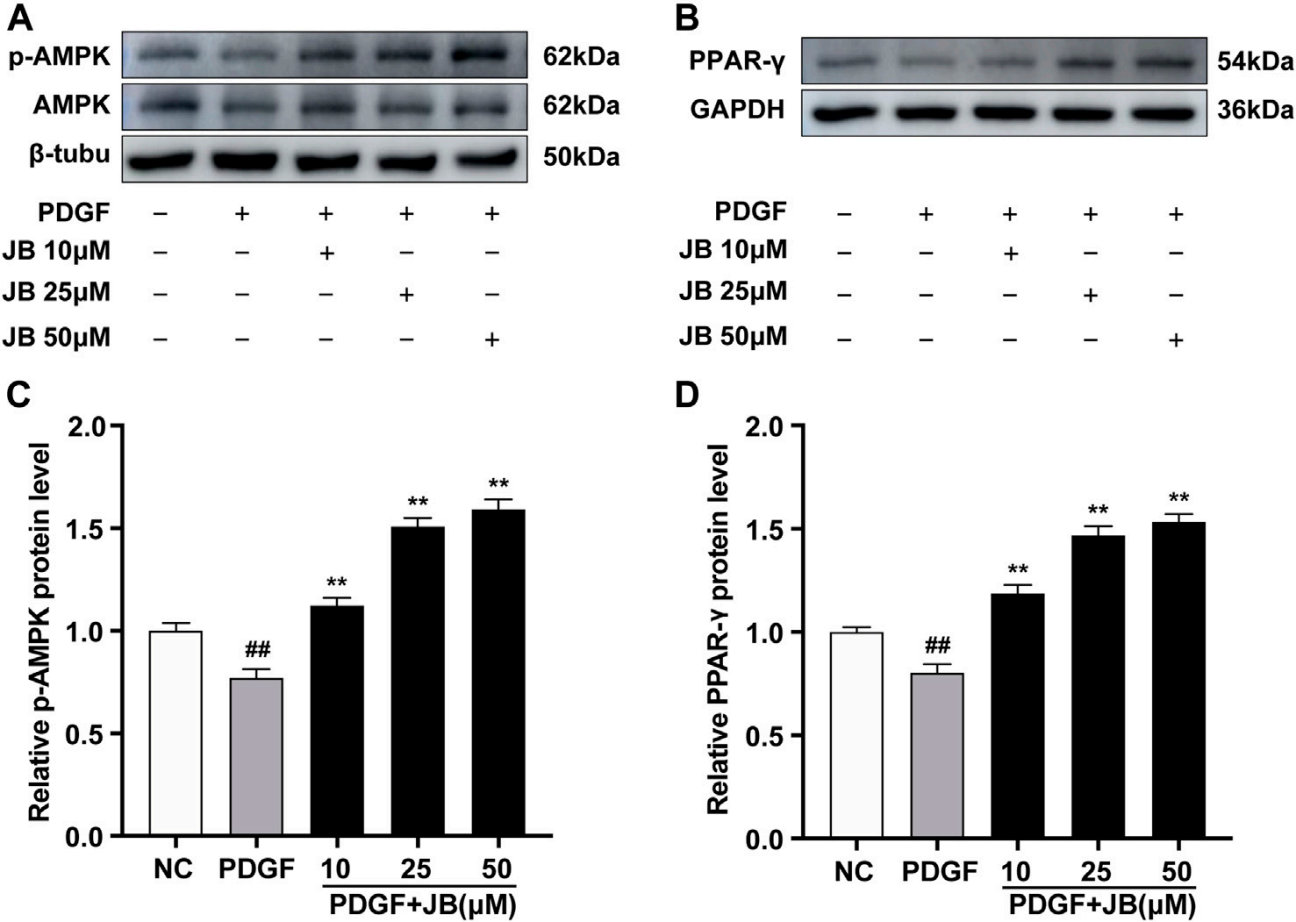
JB activated AMPK/PPAR-γ pathway in VSMCs. VSMCs were incubated with PDGF-BB in the presence of different concentrations of JB. **(A,B)** Western blotting analysis of p-AMPK, AMPK **(A)**, PPAR-γ **(B)** in VSMCs. **(C,D)** Quantification of p-AMPK, AMPK **(C)**, PPAR-γ **(D)** protein expression levels between groups. Data are expressed as mean ± S.D. ^##^ *p* < 0.01 vs. Control. **p < 0.01 vs. PDGF-BB group. *n* = 3.

To further study the mechanism of JB against VSMCs proliferation and migration, Western blot analysis was used to detect the protein expression levels of phosphorylated AMPK and PPAR-γ. In the A7r5 cultured cell model, JB (10, 25, and 50 μM) significantly enhanced the phosphorylation level of AMPK in a dose-dependent manner without changing the total level of AMPK in A7r5 (Figures 7A,C). Consistently, JB pretreatment dose-dependently enhanced PPAR-γ relative protein expression in VSMCs (Figures 7B,D).

### JB Inhibited VSMC Proliferation and Migration Through AMPK/PPAR-γ Pathway

**FIGURE 8:**
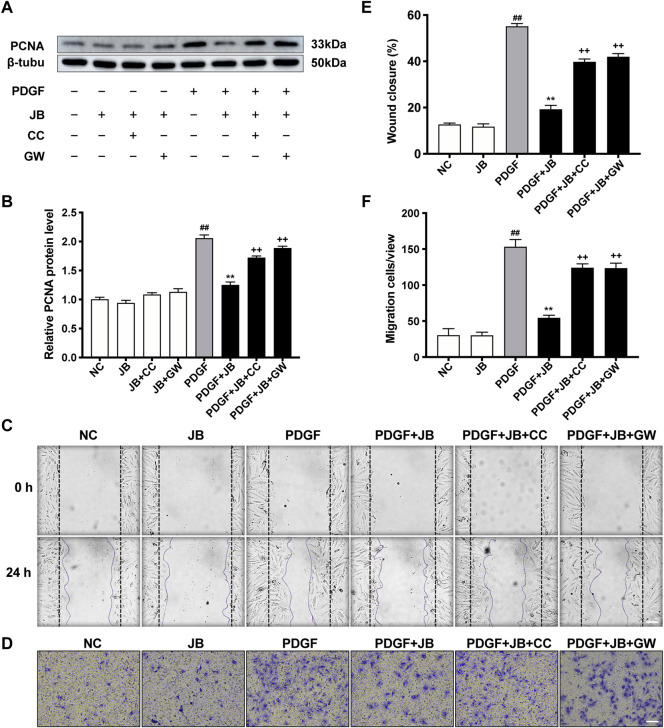
JB inhibited VSMCs proliferation and migration through AMPK/PPAR-γ pathway. VSMCs was incubated with CC or GW for 2 h and then treated with JB. **(A,B)** Western blotting analysis of PCNA in VSMCs **(A)** and corresponding protein level quantification **(B)**. **(C,E)** Representative images of cell migration by wound healing test **(C)** and quantification of wound healing in each group **(E)**. Scale bar: 100 μm. **(D,F)** Representative images of cell migration by Transwell assay **(D)** and quantification of VSMCs stained with crystal violet in each group **(F)**. Scale bar: 100 μm. Data are expressed as mean ± S.D. ^##^ *p* < 0.01 vs. Control. **p < 0.01 vs. PDGF-BB group. ^++^ *p* < 0.01 vs. the PDGF + JB group. *n* = 3.

To investigate the necessity of activated of AMPK in the alleviation of JB on PDGF-induced VSMCs proliferation and migration, we used AMPK inhibitors, compounds C (CC), and GW9662 (GW), an inhibitor of PPAR-γ. Notably, compounds C and GW9662 reversed PCNA expression (Figures 8A,B) and wound healing (Figures 8C,E). We further examined the necessity of the AMPK/PPAR-γ signaling pathway for the anti-migration ability of JB by transwell migration assay (Figure 8D to Figure 8F). Compared with the NC group, PDGF-BB induced significant migration of VSMCs, which was manifested by the positive staining of crystal violet in the larger purple area of the lower chamber. The JB treatment obviously inhibited cell migration. It is worth noting that the addition of CC and GW reversed the anti-migration activity of JB. Overall, these results suggested that JB inhibited VSMCs proliferation and migration *in vitro* by regulating the AMPK/PPAR-γ signaling pathway.

## Discussion

Vascular intervention inevitably damages the arterial intima, which leads to collagen exposure and persistent inflammation. VSMCs undergo dedifferentiation after being stimulated, that is, the phenotypic switch from the contractile phenotype to highly proliferative and migratory synthetic phenotype, which plays an essential role in vascular restenosis (Gomez and Owens, 2012). PDGF-BB is one of the most effective mitogens released from damaged blood vessels and plays an important role in promoting the proliferation of vascular smooth muscle cells. Therefore, herein, we chose PDGF-BB as an *in vitro* stimulant. Blood vessels injured by the balloon were used *in vivo* models of restenosis. Consistent with previous reports, PDGF-BB induced dedifferentiation, proliferation, and migration of VSMCs. Notably, pre-conditioning with JB in the experimental system significantly antagonized the dedifferentiation, proliferation, and migration of VSMCs induced by PDGF-BB. Consistent with *in vitro* experiments, JB significantly reduced the neointimal hyperplasia caused by balloon injury to the arteries. Further mechanistic studies have shown that its function against restenosis after the vascular intervention is mediated through AMPK/PPAR-γ pathway. Based on previous studies, animal models with restenosis after vascular intervention were established by balloon injury. Our experiments show *in vivo* that JB treatment significantly reduces the neointimal hyperplasia of balloon-damaged blood vessels, which is detected by histopathological slices with hematoxylin and eosin (HE) staining. *α*-SMA is a biomarker of VSMCs, originally expressed in the smooth muscle in the media. PCNA is an important biological indicator of cell proliferation, which has been widely used in clinical and basic research of vascular restenosis after balloon injury and considered to be related to VSMCs proliferation and migration (O’Brien et al., 1993). We tested the protein expression levels of α-SMA and PCNA in the neointima, verifying the ability of JB to inhibit the proliferation and migration of VSMCs *in vivo*.

Restenosis after the vascular intervention is an overreaction of the wound healing response after intravascular balloon injury, and endothelial reendothelialization is essential for normal wound healing (Tolva et al., 2016). Therefore, the ideal drug for the treatment of blood restenosis after vascular intervention should inhibit the proliferation of VSMCs and promote endothelial cell regeneration (Choe and Moldovan, 2017). In this study, to investigate the effect of JB on endothelial repair ability after vascular injury *in vivo*, we used Evans blue staining to examine the degree of reendothelialization of rat carotid arteries. We found that JB significantly improved the endothelial repairability of damaged arteries in SD rats.

Dedifferentiation is the first step in the proliferation and migration of VSMCs (Gomez and Owens, 2012). During the progression of restenosis after angioplasty, the enhancement of VSMC proliferation and migration ability is critical (Bennett et al., 2016). Therefore, looking for drugs that antagonize the PDGF-BB-mediated dedifferentiation, proliferation, and migration of VSMCs is a promising strategy to prevent and treat restenosis. JB is a natural saponin triterpenoid (Liu et al., 2007). Existing evidence showed that JB had significant antitumor activity on AGS human gastric cancer cells and HCT 116 human colon cancer cells (Jia et al., 2020).

Although the previous research on SZJ has focused chiefly on its neuroprotective function (Peng et al., 2000; Cao et al., 2010; Zhang et al., 2014), there are also studies suggesting that it might have cardiovascular protective functions, such as anti-platelet aggregation (Seo et al., 2013), reducing blood vessel tension (Zhao et al., 2016), and scavenging free radicals (Wu and Chen, 2019). This vascular protective ability is attractive and desirable for the potential use of this herb in the treatment or management of CVD (Fardoun et al., 2017). To our knowledge, no existing studies have reported the effects of JB on the phenotypic switch, proliferation, and migration of vascular smooth muscle. The data of this study showed that JB pretreatment could prevent PDGF-BB-induced VSMC dedifferentiation, which is confirmed by the increased expression of the VSMC synthesis gene OPN and the decreased expression of the contractile genes *α*-SMA and SM22α. Besides, in our study, JB pretreatment can inhibit PDGF-BB-stimulated VSMC proliferation and migration, and the inhibitory effect has nothing to do with apoptosis.

Autophagy plays a vital role in VSMCs, attracting more and more attention (Salabei and Hill, 2015). Autophagy induced by PDGF-BB helps to remove contractile proteins, promoting the transition of VSMCs to a synthetic phenotype (Salabei et al., 2013). And this kind of autophagy will make VSMCs tend to survive, which usually develops into excessive proliferation and migration (Salabei et al., 2013). Therefore, the regulation of autophagy might be a promising means to control the hyperproliferation of VSMCs. Previous studies have shown that 3-MA and spautin-1 inhibit VSMC autophagy and exert anti-proliferative effects (Li et al., 2012; Salabei et al., 2013). Consistently, PDGF-BB activated VSMCs autophagy in this study. Importantly, we found that JB could inhibit autophagy activated by PDGF-BB, suggesting that JB could inhibit VSMCs proliferation through autophagy activation. The key components of autophagy, such as the thiol residue of Atg4, can be directly modified by ROS, which links oxidative stress and autophagy together (Scherz-Shouval et al., 2007). PDGF-BB can cause the initial burst of free radicals or the continuous increase of oxidative stress (Sundaresan et al., 1995). The underlying mechanism of PDGF-BB activation of autophagy may involve the redox state of cells. Therefore, we speculate that autophagy is involved in PDGF-BB-induced VSMC hyperproliferation and may be related to ROS generation. In this study, consistently, PDGF-BB stimulated VSMCs to produce a large amount of ROS. It is worth noting that we found for the first time that JB inhibited PDGF-BB-induced ROS production. However, the specific regulatory mechanism between oxidative stress and autophagy in proliferative vascular diseases needs further study.

Adenosine monophosphate-activated protein kinase (AMPK) is a heterotrimeric αβγ complex, expressed in many tissues (including skeletal muscle, heart, and brain), and can act as a central regulator of energy homeostasis (Yan et al., 2018). The activation of AMPK can alleviate the formation of neointima in damaged blood vessels *in vivo* (Miyabe et al., 2014), and inhibit the phenotypic switch (Ding et al., 2016), proliferation (Wu et al., 2017) and migration (Ge et al., 2015) of VSMC *in vitro*. Many active monomers of natural plants, such as Thymoquinone (Pei et al., 2016) and Wedelolactone (Peng et al., 2017), can activate AMPK to play an anti-VSMC proliferation and migration effect. According to existing research, JB can regulate p38, JNK, RIPK1/RIPK3/MLKL, and other signal pathways (Xu et al., 2014; Jia et al., 2020). However, whether JB can regulate AMPK activity has not been reported. In this study, JB promoted the activation of AMPK. Notably, compound C (CC, an inhibitor of AMPK) could reverse the anti-proliferation and migration effects of JB on VSMCs. Peroxisome proliferator-activated receptor-γ (PPAR-γ) belongs to the nuclear receptor superfamily of ligand-activated transcription factors and has been reported to exert anti-proliferation and migration effects in many diseases (Hsueh et al., 2001; Li et al., 2017; Sanchez et al., 2018). Activation of PPAR-γ can inhibit VSMC proliferation and migration (Zhou et al., 2016). Down-regulation of PPAR-γ promotes VSMC phenotypic switch (Zhang et al., 2010), migration, and neointimal hyperplasia (Zhang et al., 2018). Previous studies reported that activation of AMPK could induce PPAR-γ (Nizamutdinova et al., 2009; Pei et al., 2016). Herein, we found that JB could induce PPAR-γ through AMPK. Furthermore, when PPAR-γ was blocked by its inhibitor GW9662, the anti-proliferation and migration effects of JB on VSMC would be subsequently inhibited.

These findings suggest that JB endows itself with anti-restenosis properties after vascular intervention through the AMPK/PPAR-γ signaling pathway. However, our animal-based and cell-based explorations are not enough to represent clinical applications, which requires further clinical verification in the future.

Overall, our results disclose for the first time that JB has anti-restenosis properties *in vivo* and *in vitro* after the vascular intervention. Specifically, JB antagonizes PDGF-BB-induced VSMC phenotypic switch, proliferation, and migration through AMPK/PPAR-γ pathway. These findings indicate that JB may be a potential clinical candidate drug for the prevention and treatment of restenosis after vascular intervention and provide a reference for further research on the prevention and treatment of vascular-related diseases.

## Data Availability

The original contributions presented in the study are included in the article, further inquiries can be directed to the corresponding author.
